# The Feasibility of Using High Resolution Genome Sequencing of Influenza A Viruses to Detect Mixed Infections and Quasispecies

**DOI:** 10.1371/journal.pone.0007105

**Published:** 2009-09-22

**Authors:** Muthannan A. Ramakrishnan, Zheng Jin Tu, Sushmita Singh, Ashok K. Chockalingam, Marie R. Gramer, Ping Wang, Sagar M. Goyal, My Yang, David A. Halvorson, Srinand Sreevatsan

**Affiliations:** 1 Department of Veterinary Population Medicine, University of Minnesota, Saint Paul, Minnesota, United States of America; 2 Department of Veterinary and Biomedical Sciences, University of Minnesota, Saint Paul, Minnesota, United States of America; 3 Biomedical Genomics Center, University of Minnesota, Saint Paul, Minnesota, United States of America; 4 Minnesota Supercomputer Institute, University of Minnesota, Saint Paul, Minnesota, United States of America; Institute of Infectious Disease and Molecular Medicine, South Africa

## Abstract

**Background:**

The rapidly expanding availability of de novo sequencing technologies can greatly facilitate efforts to monitor the relatively high mutation rates of influenza A viruses and the detection of quasispecies. Both the mutation rates and the lineages of influenza A viruses are likely to play an important role in the natural history of these viruses and the emergence of phenotypically and antigenically distinct strains.

**Methodology and Principal Findings:**

We evaluated quasispecies and mixed infections by de novo sequencing the whole genomes of 10 virus isolates, including eight avian influenza viruses grown in embryonated chicken eggs (six waterfowl isolates - five H3N2 and one H4N6; an H7N3 turkey isolate; and a bald eagle isolate with H1N1/H2N1 mixed infection), and two tissue cultured H3N2 swine influenza viruses. Two waterfowl cloacal swabs were included in the analysis. Full-length sequences of all segments were obtained with 20 to 787-X coverage for the ten viruses and one cloacal swab. The second cloacal swab yielded 15 influenza reads of ∼230 bases, sufficient for bioinformatic inference of mixed infections or quasispecies. Genomic subpopulations or quasispecies of viruses were identified in four egg grown avian influenza isolates and one cell cultured swine virus. A bald eagle isolate and the second cloacal swab showed evidence of mixed infections with two (H1 and H2) and three (H1, H3, and H4) HA subtypes, respectively. Multiple sequence differences were identified between cloacal swab and the virus recovered using embryonated chicken eggs.

**Conclusions:**

We describe a new approach to comprehensively identify mixed infections and quasispecies in low passage influenza A isolates and cloacal swabs and add to the understanding of the ecology of influenza A virus populations.

## Introduction

Influenza A virus is an enveloped RNA virus belonging to the family *Orthomyxoviridae* with a genome spanning 13.5-kilobases and consisting of eight single stranded RNA segments. The individual RNA segments range in length from 890–2341 nucleotides and encode 11 proteins [1]. There are 144 possible combinations of two surface glycoproteins, hemagglutinnin and neuraminidase, that determine the antigenic properties and subtype classification of the virus.

Influenza A viruses are zoonotic and as a group of viruses, they possess a wide host range including humans, at least 105 bird species, pigs, horses, dogs, cats, ferrets, mink and marine mammals. In the United States alone, more than 200,000 hospitalizations and 36,000 deaths annually are due to complications from seasonal influenza in humans. Globally, it is estimated that influenza causes 300,000 to 500,000 human deaths annually [2]. Multiple cases of human infections with H1N1, H5N1, H7N7, and H9N2 avian influenza viruses (AIV) have been reported since 1997, raising concerns over potential zoonosis of AIV [3].

From April 2009 a pandemic caused by a novel H1N1 virus has been ongoing. As of August 2009, there have been more than 182,000 laboratory confirmed cases of pandemic influenza H1N1, 1799 deaths, in 177 countries and territories have been reported to WHO (http://www.who.int/csr/don/2009_08_21/en/index.html). Therefore, enhanced surveillance of avian and swine influenza A viruses is necessary to provide an understanding of the ecology and evolution.

Influenza viruses have a high error rate during the transcription of their genomes because of the low fidelity of RNA polymerase [4]. The high error rate produce quasispecies, a phenomenon where many different viral genotypes co-circulate in the host, with each virus subtype potentially associated with varying levels of fitness for that host [5]. As defined by Domingo et al, “viral quasispecies are closely related (but nonidentical) mutants and recombinant viral genomes subjected to continuous genetic variation, competition, and selection” [6]. This high error rate in replication operates as a double-edged sword - improving the ability of the virus to rapidly adapt to a new host via genetic changes that aid in replication and transmission efficiency while leading to the production of defective subtypes that have reduced fitness for the current host. Some or most of these quasispecies or mixed subtypes may be missed during viral culture because a “host” (chicken embryo or cell culture) adaptation pressure [7].

The frequency of infection with multiple subtypes of the virus in wild birds or swine populations that may contribute significantly to the emergence of new viruses with altered host specificities is not known. Complete genome sequencing of influenza A viruses by the current method (RT-PCR followed by classical dye terminator chemistries) is time and resource demanding. For example, in the recent large-scale influenza sequencing project, 95 overlapping one-step RT-PCR were performed per sample to obtain the complete viral genome sequence [1]. Newly developed sequencing-by-synthesis technology has simplified the world of genomics by circumventing the need for individual segment amplification, cloning, and shotgun library preparation [8]. Pyrosequencing approach is useful for the identification of previously undetected and/or uncultured viruses [9] and has been applied in the detection of antiviral resistance markers [10], [11], [12], [13], [14], [15], [16], [17], [18], [19], detection of human virulence signatures in H5N1 [20], diagnosis [21] and sequencing of the full genome of high pathogenic H5N1 [22]. We used this de novo approach to sequence the entire genome of 10 virus isolates (eight avian influenza viruses and two swine influenza viruses) and two primary cloacal swabs. We tested the hypothesis that quasispecies and mixed infection among avian and swine influenza viruses can be identified by the new next-generation pyrosequencing.

## Results

### Pyrosequencing using Genome Sequencer FLX platform

**Figure 1:**
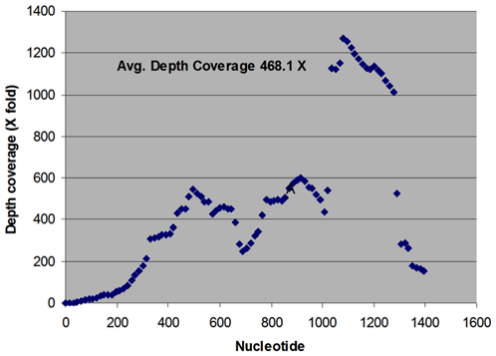
Shown is a representation of sequence coverage depth of segment 6 (NA gene) of A/mallard/South Dakota/Sg-00128/2007(H3N2) based on GSMapper (Roche, Germany). Sequence coverage varied between 2X to 1300X depending on the region of segment 6. The average redundancy of 468.1X was achieved for this segment.

Twelve samples including 10 virus isolates (eight avian influenza viruses and two swine influenza viruses propagated in embryonated chicken eggs and in MDCK cells with trypsin, respectively) and two cloacal swabs, were processed for pyrosequencing. Complete genomes (>99% Open Reading Frame) were obtained for all eight segments of each virus isolate and a cloacal swab (Table 1). For these 11 samples, the mean influenza sequence reads per small PicoTiterPlate (PTP) region was 7075, with an average read length of 232 bases. A complete ORF region (100% genome length) was obtained for five avian H3 isolates (Table 1). Both swine virus isolates yielded a clean full-length genome. For the other five virus isolates and one cloacal swab [cloacal swab of A/green-winged teal/Minnesota/Sg-00131/2007(H3N2)], 2–41 nucleotides were missing in some segments at the 3′ end but rarely at the 5′ end. Overall, ∼13400 bases (>99% of the total genome size) were covered for eleven samples with 20–787 X coverage depth. A representative coverage depth map is shown in Figure 1. For the second cloacal swab [cloacal swab of A/mallard/Minnesota/Sg-00133/2007(H4N6)], 15 influenza reads were realized with an average read length of 230 bases.

**Table 1:** Summary of the 454 sequencing results.

Sample ID	Number of PTP Region(s)	Total Influenza Reads	Avg. read Length	Segment Length Covered -Bases (Depth coverage-X)								Total Bases Covered^a^	Comments on Sequence Coverage
				PB2	PB1	PA	HA	NP	NA	M	NS		
(A/turkey/Minnesota/1138/1980(H7N3)	2 (A primer)	19122	236	2335 (378.9)	2337 (459.0)	2227 (374.4)	1726 (411.4)	1530 (138.6)	1443 (287.2)	999 (273.3)	883 (301.1)	13480	NP lacks 19 nucleotides and M lacks 2 nucleotides of coding sequences at the 3′ end
A/mallard/South Dakota/Sg-00125/2007(H3N2)	2 (1 for A and 1 for B primer)	16300	217	2335 (206.7)	2320 (138.1)	2226 (312.5)	1753 (289.6)	1557 (350.5)	1461 (506.1)	1017 (108.0)	880 (236.0)	13549	
A/northern pintail/South Dakota/Sg-00126/2007(H3N2)	2 (A primer)	21350	243	2334 (246.3)	2320 (114.7)	2226 (362.5)	1703 (466.2)	1557 (593.8)	1411 (786.9)	996 (557.6)	880 (268.6)	13427	HA lacks 14 nucleotides and NA lacks 8 nucleotides of coding sequences at the 3′ end
A/mallard/South Dakota/Sg-00127/2007(H3N2)	3 (2 for A and 1 for B primer)	9977	239	2335 (171.6)	2320 (119.0)	2226 (203.0)	1753 (182.4)	1557 (205.8)	1450 (311.7)	1017 (104.1)	865 (126.1)	13523	
A/mallard/South Dakota/Sg-00128/2007(H3N2)	3 (1 each for A, B, A+B primer)	13607	235	2335 (237.7)	2303 (91.7)	2212 (290.2)	1753 (163.7)	1557 (396.7)	1461 (468.1)	1017 (128.7)	883 (132.4)	13521	
A/green-winged teal/Minnesota/Sg-00131/2007(H3N2)	2 (A primer)	22127	242	2305 (416.9)	2321 (445.4)	2229 (387.0)	1761 (457.3)	1564 (197.8)	1447 (621.1)	1007 (262.9)	880 (472.1)	13514	
cloacal swab of A/green-winged teal/Minnesota/Sg-00131/2007(H3N2)	1 (A primer)	5341	191	2299 (55.9)	2335 (41.9)	2224 (20.1)	1728 (67.8)	1519 (102.6)	1460 (200.3)	1000 (78.6)	885 (133.4)	13450	PB2 lacks 3 nucleotides and NP lacks 12 nucleotides of coding sequences at the 3′ end
A/mallard/Minnesota/Sg-00133/2007(H4N6)	2 (A primer)	11974	245	2322 (280.5)	2317 (188.6)	2203 (169.6)	1726 (466.9)	1522 (154.5)	1451 (229.4)	985 (119.9)	871 (115.8)	13397	NP lacks 11 nucleotides and M lacks 15 nucleotides of coding sequences at the 3′ end
cloacal swab of A/mallard/Minnesota/Sg-00133/2007(H4N6)	2 (A primer)	15	-	-	-	-	-	-	-	-	-	-	Only 15 influenza reads were obtained [PB2 (2 reads), PB1 (4 reads), PA (3 reads), HA (3 reads; H1, H3, and H4), NP (1 read), NS (2 reads)]
A/bald eagle/Virginia/Sg-00154/2008(mixed) (H1N1 and H2N1 mixed isolate)	2 (1 for A and 1 for B primer)	18153	220	2282 (174.6)	2330 (100.5)	2229 (130.3)	1768 (391.9)	1545 (46.0)	1388 (64.8)	1014 (111.7)	875 (86.8)	13431 (H1N1 lineage)	PB2 lacks 20 nucleotides, NA lacks 30 nucleotides and M lacks 1 nucleotide of coding sequences at the 3′ end
				2343 (242.6)	2309 (114.6)	2222 (108.0)	1765 (288.5)	1539 (137.5)	1376 (81.9)	991 (50.1)	883 (61.8)	13428 (H2N1 lineage)	NA: lacks 41 nucleotides of coding sequences at the 5′ end
A/swine/Minnesota/SG-00239/2007(H1N2)	2 (A primer)	31329	246	2335 (568.6)	2335 (364.9)	2228 (477.7)	1769 (843.3)	1567 (528.7)	1460 (1326.9)	1025 (389.7)	830 (110.3)	13549	NS lacks 25 nucleotides of coding sequences at the 3′ end
A/swine/North Carolina/R08-001877-D08-013371/2008(H3N2)	4 (A primer)	21738	238	2357 (294.1)	2342 (210.3)	2238 (87.6)	1759 (366.3)	1564 (535.3)	1463 (1029.2)	1069 (356.2)	894 (794.6)	13686	

a Total genome size of influenza A is 13523–13645 bp (PB2 – 2341 bp; pb1 - 2341 bp, PA - 2233 bp; HA - 1728–1779 bp; NP - 1565 bp; NA - 1398–1469 bp; M - 1027 bp; NS - 890 bp).

### Comparison of sequences of cloacal swab and virus recovered using embryonated chicken eggs system reveals extensive variability

Complete genome sequences were obtained from the cloacal swab of A/green-winged teal/Minnesota/Sg-00131/2007(H3N2) and virus recovered using egg system. A comparison of sequences from each segment revealed 80–91% nucleotide identities (PB2, 90%; PB1, 87%; PA, 90%; HA, 80%; NP, 83%; NA, 82%; M, 91%; and NS, 86%) suggesting extensive variability or existence of quasispecies in the cloacal swab. In the second cloacal swab-virus isolate pair, complete sequences were obtained from the virus isolate, whereas only 15 influenza sequence reads were obtained from the cloacal swab. These 15 reads of ∼230 bp included sequences of PB2 (two reads), PB1 (four reads), PA (three reads), HA (three reads; one read each for H1, H3, and H4), NP (one read), and NS (two reads). Four sequences (one each for PB1, PA, H4, and NP) had 100% identity with the virus recovered using embryonated chicken eggs, A/mallard/Minnesota/Sg-00133/2007(H4N6).

### Comparison of Sanger sequences with pyrosequencing to resolve polymorphisms

Whole genome sequences using standard dye terminator chemistry were also available for four H3N2 viruses. Comparison of these sequences against the pyrosequencing data revealed eight single nucleotide mismatches (Table 2). Six substitutions (5 in NP and 1 in M genes) were observed in A/mallard/South Dakota/Sg-00125/2007(H3N2) isolate. Among the five NP gene polymorphisms, four were synonymous and one (A149G) led to an amino acid change (N50S). The G715A nucleotide substitution identified in the M gene of this isolate led to T239A amino acid change. A silent single nucleotide change was observed in the NP gene of A/northern pintail/South Dakota/Sg-00126/2007(H3N2) and A/mallard/South Dakota/Sg-00127/2007(H3N2) viruses. Both Sanger and pyrosequencing results were identical in all segments of A/mallard/South Dakota/Sg-00128/2007(H3N2) isolate.

**Table 2:** Comparison of the Sanger and GS FLX pyrosequencing.

Virus	Base substitution in the indicated gene^a^	
	NP	M
A/mallard/South Dakota/Sg-00125/2007(H3N2)	a149g (N50S) t441g (silent) g642a (silent) g1017a (silent) a1321c (silent)	g715a (A239T)
A/northern pintail/South Dakota/Sg-00126/2007(H3N2)	t441g (silent)	
A/mallard/South Dakota/Sg-00127/2007(H3N2)	t1191c (silent)	
A/mallard/South Dakota/Sg-00128/2007(H3N2)		

a No differences were identified in polymerase genes, HA, NA, or NS segments.

### Evidence of quasispecies in cloacal swab and virus recovered using eggs and cell culture systems

**Figure 2:**
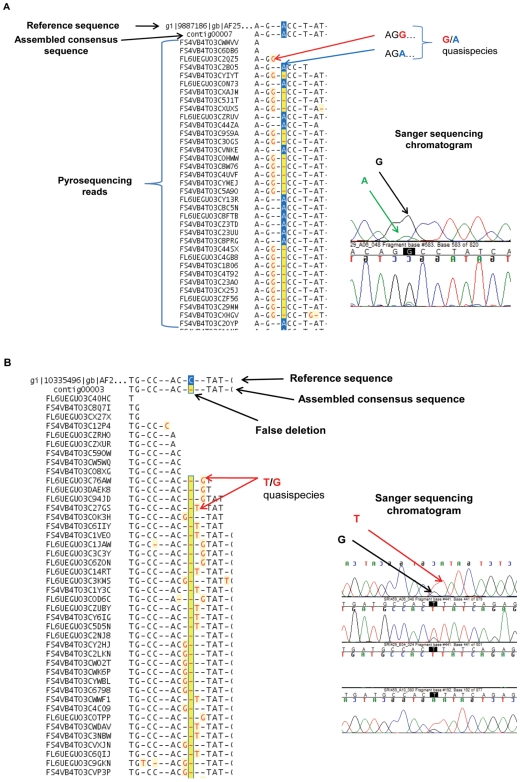
Detection of quasispecies using pyrosequencing. (A) Sequence polymorphisms in the matrix (M) gene at codon 715 of isolate: A/mallard/South Dakota/Sg-00125/2007(H3N2) is shown. The consensus sequence shows ACC (T239). However, alternate populations with GCC (A239) are present in the same position. The sequence trace of this same region generated by Sanger sequencing is also shown. This polymorphism was not called by the latter algorithm and had to be manually examined to identify the mixed peaks; (B) Polymorphisms in codon 441 of NP gene from the same isolate is shown. The consensus sequence shows a false deletion at nucleotide position 441 that was resolved by manual editing of the sequence traces. Two possible nucleotides (G and T) were identified in the same position. This polymorphism was confirmed by standard dye terminator sequencing and manual examination of chromatograms show mixed peaks at position 441 (chromatograms in both orientation are presented).

A series of genomic subpopulations or quasispecies as identified by single nucleotide polymorphisms (SNP) at specific nucleotide positions was identified in five virus isolates (four egg grown avian influenza viruses and one cell cultured swine influenza virus) and a cloacal swab. All the above samples are H3N2 subtype and all quasispecies populations observed in this study originated from mutations in NP, PB1, PA, M, and NS genes (Table 3). One example of the evidence of quasispecies in codon 715 of the M gene of A/mallard/South Dakota/Sg-00125/2007(H3N2) is shown in Figure 2A. Figure 2B shows codon 441 of the M gene of the same isolate with computational complexity of a false deletion that needed to be corrected as T/G quasispecies by manual curation. There was good agreement between polymorphic loci identified in chromatograms generated using Sanger sequencing and pyrosequencing methods. When single nucleotide polymorphisms at a particular base were present, mixed peaks were observed in Sanger's chromatogram, whereas variant populations were identified in the assembled pyrosequencing reads (Figure 2B).

**Table 3:** Co-infection and quasispecies population in different viral samples.

Sample ID	Quasispecies and position^a^					Remarks
	PB1	PA	NP	M	NS	
A/mallard/South Dakota/Sg-00125/2007(H3N2)	1725-R	423-K	149-R 441-K 642-R 1017-R 1321-M	715-R	809-R	
A/northern pintail/South Dakota/Sg-00126/2007(H3N2)	1725-R	419-Y 423-K	149-R 441-K 642-R 1017-R 1321-M	715-R	809-R	
A/mallard/South Dakota/Sg-00127/2007(H3N2)	1725-R	419-Y	149-R 441-K 642-R 1017-R 1191-Y			
A/mallard/South Dakota/Sg-00128/2007(H3N2)	1725-R		1191-Y			
cloacal swab of A/green-winged teal/Minnesota/Sg-00131/2007(H3N2)	174-Y		1021-Y 1026-R 1029-M 1125-Y 1140-R			
cloacal swab of A/mallard/Minnesota/Sg-00133/2007(H4N6)						Co-infection with three HA subtypes - H1, H3 and H4
A/bald eagle/Virginia/Sg-00154/2008(mixed)						Mixed isolate full length sequence of two clade - H1N1 and H2N2 - were obtained
A/swine/North Carolina/R08-001877-D08-013371/2008 (H3N2)	174-Y				201-R	

a Nucleotide numbering begins at each ORF; R = A/G Y = C/T M = A/C K = T/G.

### Identification of mixed infections

**Figure 3:**
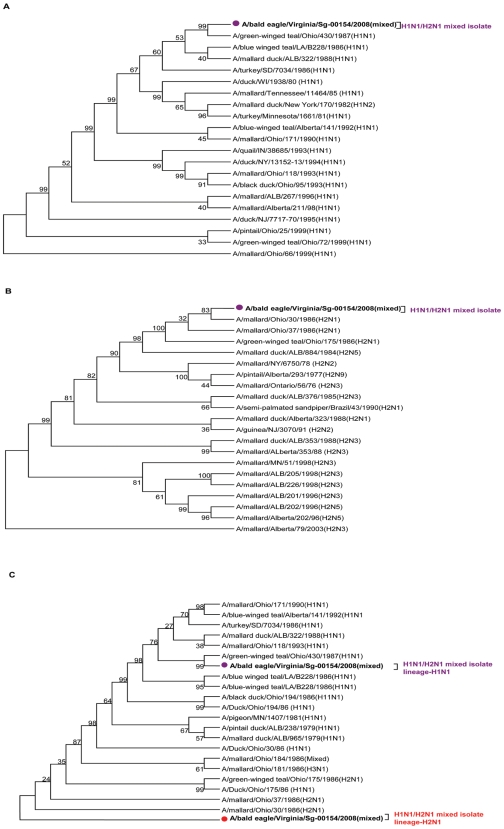
Phylogenetic analysis of HA and NA sequences from A/bald eagle/Virginia/Sg-00154/2008(H1N1/H2N1) show HA lineages of H1 (Panel 3A) and H2 (Panel 3B), and at least two lineages of N1 segment (Panel 3C). Evolutionary associations were inferred in MEGA 4.0 using the maximum parsimony algorithm with Kimura-2P correction and 1000 bootstrap replications (confidence of the branches are shown on branch bifurcations).

A bald eagle isolate, A/bald eagle/Virginia/Sg-00154/2008(H1N1/H2N1) that was originally typed by sequencing segments of HA and NA as H2N1 showed evidence of mixed subtypes by whole genome analysis. Analysis of the HA sequences from the 454 data revealed that this isolate carried both H1 (Figure 3A) and H2 (Figure 3B) subtypes, suggesting that this was a mixed infection. In addition, NA sequences of this isolate revealed two different lineages of N1 and >96% identities with each other (Figure 3C).

As described above, from 15 influenza reads (∼230 bases each) that were realized for cloacal swab of A/mallard/Minnesota/Sg-00133/2007(H4N6), there was evidence of mixed infection with H1, H3, and H4 subtypes.

## Discussion

Complete genome sequencing of influenza A viruses is essential to determine the genetic basis of pathogenicity, antiviral resistance, and understanding the evolution of viruses in a variety of hosts and environments. Previous studies on sequence-based detection of antiviral resistance and diagnostics routinely used amplification of short portion of NA or HA genes followed by pyrosequencing. Hoper et al. [22] developed the pyrosequencing protocol for complete genome sequencing of H5N1 avian influenza using locus specific PCR products. In other words, all H5N1 segments were amplified with specific primers prior to sequencing. We reasoned that segment specific amplifications would lose information regarding mixed infections or quasispecies, if present in the sample. We used a preanalytical enrichment of influenza A virus genomes from several sample types including primary samples (cloacal swabs), chicken embryo grown avian and cell cultured swine influenza viruses. Enrichment was followed by de novo sequencing to enable an unbiased realization of all possible sequences in the sample. The protocol for cDNA library generation we describe is independent of locus specific amplification primers and can be used for sequencing any unknown type of influenza A viruses. This approach is consistent with the metagenomics approaches that have helped elucidate microbial (and viral) population structures from complex matrices such as marine water, soil, feces, respiratory secretions, serum and plasma [9], [23].

Application of GS De novo Assembler or GS Reference Mapper software for our 454 sequence analysis failed to identify full-length contigs. GS assembler yielded several short contigs and GS Reference Mapper produced a few false insertion/deletions (Figure 2B). We, therefore, developed an algorithm that combines three software packages (GS De nova Assembler, GS Reference Mapper and Sequencher) to efficiently assemble the genomes and detect quasispecies. The length of genome covered was ∼13400 bases (>99% of total genome size) with high confidence at 20 to 787-X coverage. This depth coverage was sufficient for the identification of quasispecies and mixed infections.

Presence of mixed infection and quasispecies in influenza viruses has also been demonstrated by others using RT-PCR of a short segment of HA from cloacal samples [7], serial limiting dilutions of virus isolates followed by RT-PCR [24], an RT-PCR/electrospray ionization mass spectrometry (RT-PCR/ESI-MS) [25], or by serological analysis [7]. In the present study, we used pyrosequencing for the identification of mixed populations of viruses as either a viral quasispecies or co-infections with multiple strains. Finding H1, H3, and H4 in one cloacal sample [cloacal swab of A/mallard/Minnesota/Sg-00133/2007(H4N6)] indicates there was the possibility of a mixed infection in the bird from which the cloacal swab was collected compared to a clean single H4 subtype that was recovered in egg grown virus.

This is in agreement with the study of Wang et al. [7], [26] who reported up to five HA subtypes in a cloacal swab sample whereas only one HA-NA combination was recovered in isolates using embryonated eggs. If multiple strains of AIV are present in the cloacal swab, one subtype commonly outgrows the others in the aberrant host system (such as embryonated chicken eggs) while the other strains remain undetected [7], [24]. In our study, the H4 subtype may have out-competed the other two subtypes in culture. Alternately, the H4 population might be the only live virus in the sample.

A possible rationale for the relatively few influenza reads (15 reads) observed in one of the cloacal samples could be due to insufficient RNA in the original sample or RNA losses during processing for pyrosequencing. In the other cloacal swab of A/green-winged teal/Minnesota/Sg-00131/2007(H3N2), complete sequences were obtained and these sequences had 80–91% nucleic acid identities with the virus recovered using embryonated egg system. This result indicates that there was a mixed population of viruses in this cloacal swab but the H3N2 subtype possibly became the predominant subtype by out-competing other virus subpopulations in the embryonated egg system. More studies with larger numbers of matched-pair samples need to be performed to completely resolve this phenomenon.

Complete genome sequences of A/bald eagle/Virginia/Sg-00154/2008(H1N1/H2N1) showed two virus lineages (H1N1 and H2N1). Using RT-PCR based HA and NA typing, this virus was identified as H2N1. In general, unambiguous indexing of mixed subtype infections would require sequential limiting dilution, PCR, cloning, and sequencing of several clones. To our knowledge, this is the first report of full genome sequencing of all eight segments from a mixed infection representing two lineages of the virus.

In our analysis of 12 samples, quasispecies were identified from five samples (four egg grown waterfowl isolates and one cell cultured swine influenza virus). All these viruses were H3N2 and identified quasispecies originated from mutations in NP, PB1, PA, M, and NS genes but not in HA, NA or PB2 genes. The four waterfowl isolates used in our study were recovered at the same study site and on the same day. This result concurs with the study of Dugan et al., [24] in which quasispecies were identified among H4N6 isolates that were recovered at the same study site, from the same species (mallard), and on the same day.

Inasmuch as the mutation rate for type A influenza viruses is estimated at one nucleotide change per 10,000 nucleotide during replication and most infections are caused by as many as 10 to 1000 virions which likely possess varying numbers of nucleotide differences in their genomes, one can expect that each influenza A virion is possibly a quasispecies. However, we identified relatively few quasispecies - probably because the currently available sequence analysis software do not allow robust quasispecies analysis and extensive manual curation is necessary. We believe that with the help of improved bioinformatic tools we would detect more quasispecies populations in our sample sets.

The method described in the current study does not require virus propagation, sequence information and circumvents the need for cloning and library construction prior to sequencing. Thus the currently described method is simple and less time consuming compared to Sanger sequencing. Despite these obvious advantages the cost of equipment is high and requires extensive bioinformatic expertise for assembling and analysis of the contigs.

In conclusion, using an unambiguous genome sequencing approach, we present evidence of quasispecies and mixed infections among influenza A viruses that could help shape our understanding of the ecology and evolution of these viruses. Future studies should be undertaken to - 1) strengthen the interpretation of culture and sequence data generated by current influenza A virus surveillance networks; 2) establish novel influenza sequence-based evolutionary analyses; and 3) provide an improved understanding of influenza subtype stability and transmission in a wide array of mammals and birds.

## Materials and Methods

### Virus samples

Twelve samples, including eight avian influenza viruses grown in embryonated chicken eggs, two swine influenza viruses propagated in MDCK cells with trypsin, and two influenza A virus positive cloacal samples were used: 1) A/mallard/South Dakota/Sg-00125/2007(H3N2), 2) A/northern pintail/South Dakota/Sg-00126/2007(H3N2), 3) A/mallard/South Dakota/Sg-00127/2007(H3N2), 4) A/mallard/South Dakota/Sg-00128/2007(H3N2), 5) A/green-winged teal/Minnesota/Sg-00131/2007(H3N2), 6) A/mallard/Minnesota/Sg-00133/2007(H4N6), 7) A/bald eagle/Virginia/Sg-00154/2008(H1/H2N1) (mixed isolate), 8) A/swine/Minnesota/Sg-00239/2007(H1N2), 9) (A/turkey/Minnesota/1138/1980(H7N3), 10) A/swine/North Carolina/R08-001877-D08-013371/2008 (H3N2), 11) cloacal swab of A/green-winged teal/Minnesota/Sg-00131/2007 (H3N2), and 12) cloacal swab of A/mallard/Minnesota/Sg-00133/2007(H4N6). All avian isolates were grown in embryonated chicken eggs while swine viruses were grown in Madin Darby canine kidney (MDCK) cells with trypsin. All isolates were passaged once or twice only.

### RNA extraction and enrichment of influenza RNA segments

Total RNA was extracted from allantoic fluid/cell culture/cloacal swab using QIAamp Viral RNA Mini kit (Qiagen) as per the manufacturer's instructions. To reduce the contaminating host nucleic acids commonly observed in viral RNA preparations, viral RNA molecules were captured and enriched through the hybridization of a biotin-labeled oligonucleotide directed to the conserved 5′-end of all eight segments of influenza A virus genome. Total RNA (50-µL; ∼50-ng/µL) was incubated in the presence of 200-µL of 6X SSPE buffer containing 0.1 units/µL of SUPERase-In (Ambion) and 0.5 µM of the 5′-Capture Oligo (5′-CCT TGT TTC TAC T-biotin-3′) at 70°C for 5 minutes followed by 15 minutes at 39°C. Equal volume (240-µL) of 2X binding and washing buffer containing 0.5 mg of washed Dynabeads MyOne Streptavidin C1(Invitrogen) was added to the above RNA samples and mixed thoroughly with a pipette. Fifty micro liters (a total of 0.5 mg) of Dynabeads MyOne Streptavidin C1 beads were washed with 1X binding and washing (B&W) buffer as per the manufacturer's instructions and resuspended in 240-µL of 2X B&W buffer (10 mM Tris-HCL pH 7.5, 1 mM EDTA, 2 M NaCl, 0.1% Tween 20). The sample was incubated at room temperature for 30 min. with gentle shaking in the orbital shaker and then placed on a magnetic stand for 3 min. The supernatant was removed by aspiration with a pipette and the coated beads were washed four times with 1X B&W buffer. The captured RNA was eluted from the beads by incubating at 65° C for 5 min with 40-µL of 10 mM EDTA, pH 8.2, in 99% formamide. The tube was placed on the magnetic stand for 3 min. and the supernatant, containing enriched RNA, was aspirated with a pipette.

### Fragmentation of enriched RNA and cDNA synthesis

The enriched viral RNA was fragmented into a size range compatible with sequencing on the Genome Sequencer FLX. Five micro liters of 5X RNA Fragmentation Buffer (200 mM Tris-acetate, pH 8.1, 500 mM Potassium acetate, 150 mM Magnesium acetate) was added to 20-µL of enriched viral RNA. The samples were mixed thoroughly by pipeting, incubated for 2 min at 82°C, and then immediately transferred to ice to stop the fragmentation reaction. The reaction volume was increased to 50-µL by adding RNase free water, purified with RNAClean (Agencourt) as per the manufacturer's instructions and eluted with 20-µL of RNase free water.

The fragmented RNA sample was reverse transcribed in 20-µL final volume using random hexamer (5′-phosphate-NNNNNNN-3′) and Superscript First-Strand Synthesis System for RT-PCR (Invitrogen) as per the manufacturer's instructions. Each reaction consisted of 7-µL of fragmented RNA and 2-µL of 500-µM primers. After reverse transcription, the RNA was removed by hydrolysis by adding 20-µl of Denaturation Solution (0.5 M NaOH, 0.25 M EDTA) and incubating at 65°C for 20 minutes. The mixture was neutralized by adding 20-µl of 0.5 M HCI in 0.5 M Tris-HCl, pH 8.0. The resultant sscDNA was recovered with RNAClean (Agencourt) as per the manufacturer's instructions and eluted from the beads with 20-µL of RNase free water.

### Adapter Ligation

For clonal amplification and sequencing on the Genome Sequencer FLX, the sscDNA required the addition of adaptors to each terminus. The adaptors have been designed to enforce directional ligation to the sscDNA, such that one will be uniquely ligated to the 5′-end (sscDNA Adaptor A) and the other to the 3′-end (sscDNA Adaptor B) of the sscDNA. Each adaptor is comprised of two complimentary oligonucleotides that are annealed together as described. The 3′-end adaptor consists of “sscDNA Oligo B” (5′-biotin- GCCTTGCCAGCCCGCTCAGNNNNNN-phosphate- 3′) and “sscDNA Oligo B-prime” (5′-phosphate- CTGAGCGGGCTGGCAAGG-dideoxyC-3′) which, after annealing, results in “sscDNA Adaptor B” with a 3′-random overhang of six nucleotides. Similarly, the 5′-end adaptor consists of “sscDNA Oligo A-prime” (5′-NNN NNN CTG ATG GCG CGA GGG AGG dideoxyC-3″) and “sscDNA Oligo A” (5′-GCCTCCCTCGCGCCATCAG-3′) which form “sscDNA Adaptor A” with a six nucleotide 5′-end overhang. The adapter ligation reaction was carried out using T4 DNA ligase (New England Biolabs) in a total volume of 30-µL, containing 3 µL of 10X ligase buffer, 1-µL of (1.67 µM final conc.) adapter A, 1-µL of (6.67 µM final conc.) adapter B, 5-µL (2000 cohesive end units) of T4 DNA ligase, 15-µL of sscDNA and 5-µL of water. The reaction mixture was incubated at RT for 2 hrs; the ligated sscDNA was recovered with Dynabeads MyOne Streptavidin C1 (20-µL beads per sample) and eluted by incubating at 65°C for 5 min with 40-µL of 10 mM EDTA, pH 8.2, in 99% formamide. The final sscDNA was purified with two rounds of RNAClean (Agencourt) and eluted in 20-µL of nuclease free water.

The final adapted sscDNA was amplified using Advantage 2 PCR Kit (Clontech) in a total volume of 50-µL containing 5-µL of 10X Advantage 2 buffer, 2-µL of 50X dNTP mix (10 mM each), 10-µL (10 µM) Primer A (5′-GCC TCC CTCGCG CCA-3′), 10-µL (10 µM) Primer B (5′-GCC TTG CCA GCC CGC-3′), 1-µL of 50X Advantage polymerase mix, 10-µL of sscDNA, and 12-µL of nuclease free water. The PCR conditions used were: 96° C for 4 min; 30 cycles of 94° C for 30 s and 64° C for 30 s; 68° C for 3 min; hold at 14° C. The PCR product was purified with two rounds of AMPure (Agencourt) as per the manufacturer's instructions. The double stranded DNA library was eluted with 20-µL of water and quantified with the Quant-iT Picogreen dsDNA Assay Kit (Invitrogen). Emulsion PCR amplification was carried out using either primer A or primer B or both for bidirectional sequencing. The sequencing reactions were carried out in small regions of the PicoTiterPlate (1–4 regions/sample) on the Genome Sequencer FLX (GS FLX) platform.

### Sequencing data analysis

Data analyses were performed on the Linux servers or Windows work station at the Minnesota Supercomputing Institute. All the sequencing reads were blasted against influenza genome in NCBI blast version.2.2.16. The ‘non influenza’ sequences were filtered out and only influenza reads were assembled in GS De nova Assembler Version 2.0.00.20 and mapped in GS Reference Mapper Version 2.0.00.20. The influenza contigs obtained using the above software were reassembled in Sequencher Version 4.8 (Genecodes).

### Quasispecies identification

All the influenza reads were run in GS De novo Assembler with three sets of parameters: minimum overlap (MOL) of 40 nucleotides and 90% identity, MOL of 100 and 100% identity, and MOL of 200 and 100% identity. The larger contigs (>500 bases) obtained by the above method were BLAST analyzed using NCBI resources and the most closely related sequences, referred to as reference sequences, for each segment were downloaded. All the influenza reads were mapped with reference sequences in GS Reference Mapper. The contigs obtained from GS Assembler and the consensus sequences obtained from GS Mapper were reassembled in Sequencher 4.8. The new contigs were then examined for ambiguous bases (e.g. R, Y, K etc.) and particular base positions were manually examined for the presence of more than one kind of base (quasispecies) in GS Reference Mapper.

### Comparison of Sanger and pyrosequencing

All the eight segments of four AIV isolates - A/mallard/South Dakota/Sg-00125/2007(H3N2), A/northern pintail/South Dakota/Sg-00126/2007(H3N2), A/mallard/South Dakota/Sg-00127/2007(H3N2), A/mallard/South Dakota/Sg-00128/2007(H3N2) were sequenced by classical Sanger sequencing method using ABI PRISM 3730xl DNA Analyzer (ABI) and the results were compared with the consensus sequences of pyrosequencing obtained with GS Reference Mapper software.
